# Orientation Maps in Mouse Superior Colliculus Explained by Population Model of Non-Orientation Selective Neurons

**DOI:** 10.1523/JNEUROSCI.1133-25.2025

**Published:** 2025-11-05

**Authors:** Austin Kuo, Justin L. Gardner, Elisha P. Merriam

**Affiliations:** ^1^Laboratory of Brain and Cognition, NIMH, NIH, Bethesda, Maryland 20892; ^2^ Neurosciences Interdepartmental Program, Stanford University, Stanford, California 94305; ^3^Wu Tsai Neurosciences Institute, Stanford University, Stanford, California 94305; ^4^Department of Psychology, Stanford University, Stanford, California 94305

**Keywords:** computational modeling, neural populations, orientation, spatial frequency, superior colliculus, visual neuroscience

## Abstract

Mouse superficial superior colliculus (sSC) has been found to have orientation selective maps, suggesting a fundamentally different selectivity than in primate SC. Moreover, orientation selectivity in mouse sSC appears to change with stimulus properties such as size, shape, and spatial frequency, in contradistinction to the computational principle of invariance in primates. To reconcile mouse and primate mechanisms for orientation selectivity, we constructed a computational model of mouse sSC populations with circular-symmetric, center-surround (i.e., not intrinsically orientation selective), stimulus-invariant receptive fields (RFs), classically used to describe monkey lateral geniculate nucleus (LGN) neurons. This model produced population maps similar to those found in mouse sSC, which show strong radial orientation preferences at retinotopic locations along stimulus edges. We show how this selectivity depended critically on spatial frequency tuning of the model units. The model predicted a shift from radial to anti-radial orientation preferences from the same simulated units at high stimulus spatial frequencies, also consistent with measurements from mouse sSC. We found intrinsically oriented RFs were largely unnecessary to explain the imaging data but could explain a possible small subpopulation of intrinsically orientation selective neurons. We conclude that to study orientation selectivity in mouse sSC and other systems, the problem is not the choice of stimulus. Rather than endless tweaks to find the perfect, unbiased stimulus, image-computable population modeling is the solution. Regardless of the stimulus presented, comparing how well models of intrinsically or non-intrinsically orientation selective units account for empirical data provides definitive evidence for underlying neural selectivity.

## Significance Statement

Measurements of neural population activity from mouse superior colliculus (SC) show patterns of orientation selectivity differing markedly from those observed in primates. Do such measurements necessarily imply different neural mechanisms across species? We developed a modeling framework that explicitly predicts population activity using well-established mechanisms from classic primate single-unit neurophysiology. Notably, this framework was sufficient to explain a diverse array of population measurements in mouse SC. Our results reconcile seemingly contradictory neural phenomena across species and visual areas through a principled approach for making inferences across measurement scales (i.e., single neurons to neural populations), providing a unifying framework for determining shared computational mechanisms broadly throughout the brain.

## Introduction

A foundational finding of sensory neurophysiology is that selectivity for basic stimulus properties is invariant to different stimulus manipulations and behavioral states. A prominent example of this is orientation selectivity in V1. Orientation selectivity arises from feedforward anatomical projections from the LGN to visual cortex (Hubel and Wiesel, 1962; De Valois et al., 1982; Reid and Alonso, 1995; Ferster et al., 1996). The resulting neural selectivity is invariant to changes in stimulus contrast (Sclar and Freeman, 1982; Skottun et al., 1987; Liu et al., 2018), presence of suppressive stimuli (Carandini et al., 1997), and attentional state (McAdams and Maunsell, 1999). This extensive line of research suggests that selectivity can be considered a fixed property that does not change with stimulus context.

A recent study (Liang et al., 2023) found orientation selectivity depended on the visual stimulus. Ca^2+^ imaging measurements from mouse superficial superior colliculus (sSC) neurons demonstrated maps of orientation selective responses localized at retinotopic locations corresponding to stimulus edges. Furthermore, orientation selective responses at the same locations along the stimulus edge shifted to show preference for orthogonal orientations with increasing stimulus spatial frequency. These changes in selectivity with stimulus properties might suggest that the computational principles underlying receptive field (RF) properties known from primate research are not shared by mouse SC.

Here we ask whether the changing orientation selectivity reported in mouse sSC can be reconciled with fixed RF properties. We simulated neural responses through a computational model to determine whether Liang et al.’s (2023) results necessarily imply that mouse sSC neurons have RFs that change their intrinsic orientation selectivity. We constructed a simple spatial stimulus energy model of mouse sSC using RFs with fixed properties based on previously reported measurements (Gardner et al., 1999; Wang et al., 2010; Inayat et al., 2015; Kasai and Isa, 2016; Ito et al., 2017; De Franceschi and Solomon, 2018). We found that a model built entirely of fixed RFs was sufficient to reproduce the phenomenon of changing orientation selectivity.

We found that intrinsically orientation selective RFs were not necessary for population orientation maps. Rather, circular-symmetric, center-surround RFs were sufficient to simulate a wide range of population-scale observations in mouse sSC (Ahmadlou and Heimel, 2015; Feinberg and Meister, 2015; Liang et al., 2023). The modeling framework does not necessarily preclude the existence of RFs with intrinsically oriented structure in mouse sSC, as we found that simulated V1-like RFs could also produce changing population orientation preference maps and were needed to explain a small population of preference-maintaining neurons from the imaging data (Liang et al., 2023). As described in this study, many of the changes in orientation selectivity with spatial frequency are readily explained by considerations of both stimuli and RFs in the spatial frequency domain. These results highlight that spatial frequency selectivity is essential for producing population orientation maps and the phenomenon of changing orientation selectivity.

## Materials and Methods

### Overview of simulations

**Figure 1.**
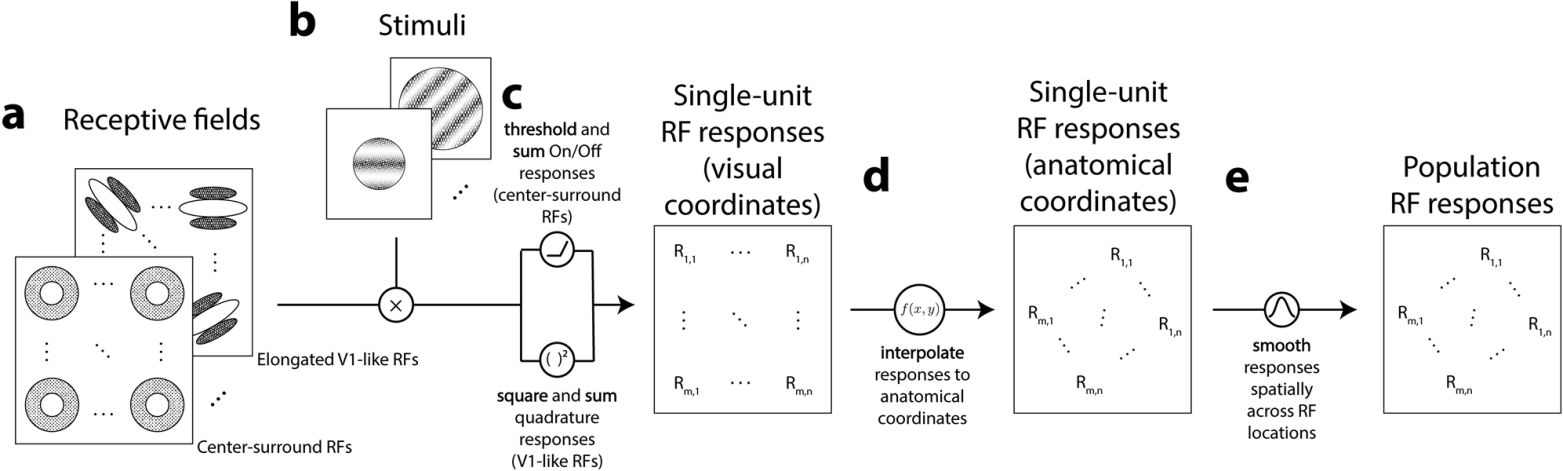
Model of single-unit and population responses from simulated RFs. ***a***, Center-surround and randomly oriented V1-like RFs tiled a dense grid of locations; the grid of RF centers extended beyond the extent of all stimuli. ***b***, Stimuli were sinusoidal gratings with varying orientations, phases, and spatial frequencies; specific parameters depended on simulation. Stimulus apertures varying in size and shape matched those used in Liang et al. (2023). ***c***, Stimuli and RFs were pointwise multiplied for each pairwise combination of stimulus and RF parameters and combined across RF On-Off pairs or RF phase to generate single-unit RF responses. ***d***, RF responses for evenly sampled locations in anatomical space were estimated from grid of RF responses in visual coordinates using natural neighbor interpolation. ***e***, Single-unit RF responses in anatomical space were spatially smoothed using a circular symmetric Gaussian kernel, resulting in a set of simulated population RF responses.

We simulated several experiments with a range of stimuli and with two different RF structures to highlight how population maps and changes in orientation selectivity found through Ca^2+^ imaging could be captured by models of circular-symmetric, center-surround, and elongated subfield (V1-like) RFs. Simulated neurons with center-surround and V1-like RF structure tiled a grid of coordinates in visual space (Fig. 1*a*). Simulated responses from these RFs were obtained (Fig. 1*c*) by presenting simulated RFs with grating stimuli that matched those used to obtain the imaging data from Liang et al. (2023) (Fig. 1*b*). The grid of RF responses in visual coordinates was then used to interpolate RF responses in anatomical coordinates, matching the coordinate space of the imaging data (Fig. 1*d*). We simulated population RF responses by applying a spatial smoothing procedure on our simulated single-unit RF responses in anatomical coordinates (Fig. 1*e*). The main simulations we conducted are briefly described here, with details of simulation and stimuli presented in the sections below. All simulations were performed using code written in Matlab 2023b and are available at https://github.com/austinchkuo/mousesc-ori-model.

### Aperture shape simulation (Fig. 3): manipulation of stimulus aperture shape and size

Population orientation selectivity was found in mouse sSC to change with both size and shape of stimulus apertures. We modeled these changes in orientation selectivity using circular-symmetric, center-surround RFs which had no intrinsic orientation preference.

### Spatial frequency simulation (Figs. 4 and 5): manipulation of stimulus spatial frequency

Population orientation selectivity was found in mouse sSC to change with the spatial frequency of the stimulus. We modeled these changes in orientation selectivity using circular-symmetric, center-surround RFs. We performed a grid search to find center-surround RF parameters providing the best qualitative fit to the imaging data (Fig. 5).

### Bar stimulus simulation (Fig. 6): manipulation of stimulus type

In addition to oriented gratings, other imaging experiments observed patterns of orientation selectivity using oriented bar stimuli (Feinberg and Meister, 2015; Inayat et al., 2015; de Malmazet et al., 2018; Kasai and Isa, 2022; Li and Meister, 2023). We simulated RFs with circular-symmetric, center-surround structure to test whether bar stimuli could produce population orientation preferences similar to those found using oriented grating stimuli.

### RF properties simulation (Figs. 7 and 8): manipulation of RF structure at single unit and population scales

Calcium imaging measurements in mouse sSC demonstrated changes in orientation selective responses to grating stimuli with different apertures at both the population and single-unit scale. We simulated RFs with circular-symmetric, center-surround structure and with elongated subfields (V1-like RFs) to compare changes in predicted orientation preferences for both simulated RF types at single-unit and population scales.

### RF models (Fig. 1a)

#### Center-surround RFs

Center-surround RFs were constructed by taking the difference of two, 2D circular-symmetric Gaussian functions in the spatial domain, resulting in a difference-of-Gaussians RF, defined by the following equation:

$$f(x,y)=e^{-\left(\frac{(x-x_{o}{)}^{2}}{2\sigma_{\mathrm{center}}^{2}}\;+\;\frac{(y-y_{o}{)}^{2}}{2\sigma_{\mathrm{center}}^{2}}\right)}-CS_{\mathrm{ratio}}e^{-\left(\frac{(x-x_{o}{)}^{2}}{2\sigma_{\mathrm{surr}}^{2}}\;+\;\frac{(y-y_{o}{)}^{2}}{2\sigma_{\mathrm{surr}}^{2}}\right),}\;(1)$$

where *x*_o_ and *y*_o_ denote the center locations of the Gaussians; *σ*_center_ and *σ*_surr_ denote the standard deviation, or width, of the center and surround Gaussians; and CS_ratio_ denotes the ratio of surround to center amplitude, specifically the volume under the surround Gaussian to the volume under the center Gaussian. In the aperture shape simulation (Fig. 3), *σ*_center_ was 1.5°, *σ*_surr_ was 11.4°, and CS_ratio_ was 0.8. In the spatial frequency simulation and RF properties simulation, *σ*_center_ was 0.8°, *σ*_surr_ was 4.7°, and CS_ratio_ was 0.4. We systematically studied the effect of changing these parameters in the grid search corresponding to the spatial frequency simulation (Fig. 5). The values used were *σ*_center_ = 0.8°, *σ*_surr_ = {3, 4.7, 5.2, 5.5, 5.7°}, and CS_ratio_ = {0.2, 0.4, 0.6, 0.8, 1}. Additionally, the grid search also included a condition with no surround, corresponding to *σ*_center_ = 0.8, CS_ratio_ = 0. In the bar stimulus simulation, we used RF sizes of *σ*_center_ = {0.8,3,6°}, *σ*_surr_ = {4.7,6.5,15°}, and CS_ratio_ = 0.4.

A pair of difference-of-Gaussians functions with inverted polarity was created for each location to simulate both On-center and Off-center RFs. The responses from the On-center and Off-center RFs were subsequently thresholded and summed to obtain a single On/Off pair response at each RF location (Fig. 1*c*). This procedure does not qualitatively impact the results of the study but was done simply to produce responses invariant to the polarity of the stimulus (bright/dark vs dark/bright).

For the aperture shape simulation, we modeled RFs with a spatial frequency preference of 0.04 cycles/° (*σ*_surr_ = 11.4°, *σ*_center_ = 1.5°, CS_ratio_ = 0.8), following RF sizes and spatial frequency preferences reported in the literature (Wang et al., 2010; De Franceschi and Solomon, 2018). In all subsequent simulations, we used center-surround RFs, with *σ*_center_ = 0.8°, *σ*_surr_ = 4.7°, and CS_ratio_ = 0.4, following a grid search (Fig. 5) for center-surround RF parameters that produced the best qualitative match to empirical data in the spatial frequency simulation (Fig. 4). These parameters yielded RFs with a center (i.e., preferred) spatial frequency of 0.08 cycles/°. This spatial frequency preference was determined by slicing the Fourier representation of the RF along an axis through the origin to obtain a one-dimensional Fourier spectrum and recording the spatial frequency corresponding to the peak amplitude.

To simulate the spatial distribution of RF centers across mouse sSC, we created a densely tiled grid of RF locations in visual field coordinates. RF center coordinates *x_o_* and *y_o_* ranged from −30 to 30°, in 0.25° steps (58,081 RF locations) when calculating responses to circular aperture stimuli with radii of 20°, as well as square and diamond aperture stimuli (Fig. 3*a*, top row, far left, center right, far right). We extended the range of both *x_o_* and *y_o_* to ±40° (103,041 RF locations) and ±50° (160,801 RF locations) when calculating responses to stimuli that had radii of 30° (Fig. 3*a*, top row, center left) and 40° (Fig. 3*a*, top row, center). In the bar stimulus simulation (Fig. 6), we simulated a grid of RFs with RF center coordinates *x_o_* and *y_o_* ranging from −20 to 20°, in 1° steps (1,681 RF locations).

When simulating preference-switching and preference-maintaining neurons (Fig. 8), we matched 53 simulated single-unit RF locations to the 53 single-unit locations reported in the imaging data, with RF centers falling within the central 20°×20° square in visual space [Fig. 8*a–c*, left column; adapted from Liang et al. (2023), their Fig. 4a–c]. In simulation, the distribution of these RF center locations in anatomical space corresponded to approximately −2.5 to −6.5° from the center of visual space in both *x*- and *y*-dimensions.

RFs and stimuli were simulated in a 200 × 200 pixel grid representing a −60 to 60° field of view (FOV). Although the pixel sampling may appear sparse, the maximum preferred spatial frequency of the simulated RFs was only 0.08 cycles/°, and the highest stimulus spatial frequency used was 0.32 cycles/°, both of which were well below the Nyquist frequency of 0.83 cycles/° for this FOV. In the bar stimulus simulation (Fig. 6), the FOV encompassed −30 to 30° in a 1,200 × 1,200 pixel grid.

#### Elongated subfield (V1-like) RFs

V1-like RFs were constructed by pointwise multiplying a 2D sinusoidal grating with an elongated 2D Gaussian envelope. The 2D grating was defined by the following equation:

$$f_{g}(x,y)=\cos(2\pi \nu_{g}\cos(\theta_{g})x+2\pi \nu_{g}\sin\;(\theta_{g})\;y+\phi_{g}),\;(2)$$

where *v_g_* denotes the spatial frequency of the grating in cycles/°, *θ_g_* denotes the orientation of the grating in radians, and *ϕ_g_* denotes the phase of the grating in radians.

Note that for the intuitive convention of defining a horizontal grating as 0° and increasing angle as a counterclockwise rotation (thus aligning orientation with polar angle), the following conversion was made:

$$\theta_{g}=-\frac{\pi (\theta_{c}+90)}{180},\;(3)$$

where *θ_c_* denotes orientation (in degrees) in the described convention. Throughout this study, all descriptions of grating orientation are made with respect to *θ_c_*. The shape of the Gaussian envelope was defined as follows:

$$f(x,y)=e^{-\left(\frac{(x-x_{o}{)}^{2}}{2\sigma_{\mathrm{maj}}^{2}}\;+\;\frac{(y-y_{o}{)}^{2}}{2\sigma_{\min}^{2}}\right)},\;(4)$$

where $\sigma_{\mathrm{maj}}$ and $\sigma_{\min}$ denote the standard deviations of the major and minor axes and *x_o_* and *y_o_* denote the location of the RF center. The values of the envelope were then rescaled to range between 0 and 1. The lengths of the minor and major axes were defined as follows:

$$\sigma_{\min}=\frac{n_{\mathrm{subregions}}}{4\sqrt{2}f_{\mathrm{pref}}},\;(5)$$

$$\sigma_{\mathrm{maj}}=\frac{LW_{\mathrm{ratio}}}{n_{\mathrm{subregions}}}\sigma_{\min},\;(6)$$

where *n*_subregions_ refers to the number of excitatory plus inhibitory subregions in the RF, *f*_pref_ refers to the optimal stimulus frequency for the RF (by definition, the frequency of the grating), and LW_ratio_ refers to the ratio of the major to minor axis lengths. Here, we chose *f*_pref_ to be 0.08 cycles/°, to match the preferred spatial frequency obtained from the grid search described for the center-surround RFs (Fig. 5). Prior modeling work of RF properties in cat striate cortex has suggested estimates of *n*_subregions_ and LW_ratio_ as 2 and 4 to be reasonable assumptions (Gardner et al., 1999). The Gaussian envelope was rotated along with the grating so that the major axis was always aligned parallel to the grating.

The spatial tiling of V1-like RFs was defined by RF center coordinates *x_o_* and *y_o_* ranging ±50°, in 0.25° steps (160,801 RF locations). At each RF location, eight oriented RFs across equally spaced orientations $\left(\theta_{c}=0,\;22.5,\;45,\ldots ,\;157.5^{\circ }\right)$ were constructed. For each RF orientation, we generated two RFs in quadrature phase $\left(\phi_{g}=0,\;90^{\circ }\right)$, which were later used to obtain phase-invariant responses. Such responses simulated those in complex cells by squaring and summing responses of quadrature pair RFs (Adelson and Bergen, 1985; Fig. 1*c*). This procedure produces stimulus phase-invariant responses. We note that averaging simple cell RF responses (as opposed to the quadrature pairing we describe here) across stimuli of many randomized phases will achieve qualitatively similar results at the cost of computational efficiency.

The spatial locations of V1-like RFs used to simulate preference-switching and preference-maintaining neurons (Fig. 8) were the same as previously detailed for center-surround RFs. *θ*_c_ (RF orientation angle) for each of the 53 units was chosen to match the orientation preferences reported in the imaging data (Fig. 8*c*, middle-left panel), instead of randomly chosen as in all other simulations of V1-like RFs.

### Stimuli (Fig. 1b)

#### All simulations

Stimuli were 2D static sinusoidal gratings presented in several aperture types with hard boundaries, with the gratings being defined as in Equations 2 and 3.

Twelve grating orientations $\left(\theta_{c}=0,\;15,\;30,\ldots ,\;165^{\circ }\right)$ and four grating phases $\left(\phi_{g}=0,\;90,\;180,\;270^{\circ }\right)$ were used to probe orientation selectivity. Though the simulated RFs in this study were designed to be phase-invariant, multiple stimulus phases were chosen to minimize potential response differences from numerical error. Gratings were presented at 100% contrast on a gray background. Minimum luminance was assigned a value of −1, maximum luminance was assigned a value of 1, and the gray background was assigned 0. The FOV of the entire stimulus was constructed to be 120° × 120° in visual field coordinates, corresponding to a sampling area of 200 × 200 pixels. Simulated stimuli never extended beyond this FOV.

#### Aperture simulation

Circular apertures had a radius of either 20, 30, or 40°. Square or diamond (square rotated 45°) apertures had a side length of 40°. Spatial frequency of the gratings *v_g_* was 0.04 cycles/° (Fig. 3*a*, top row).

#### Spatial frequency simulation

Circular apertures had a radius of 30°. Grating frequencies were chosen from six possible spatial frequencies $\left(\nu_{g}=0.01,\;0.02,\;0.04,\;0.08,\;0.16,\;0.32\;\mathrm{cycles}{/}^{\circ }\right)$ (Fig. 4*a*, top row).

#### Bar stimulus simulation

Bar stimuli had a width of 2° and were either horizontally or vertically oriented. Bar center locations ranged from −30 to 30° in 0.25° steps in *x*- and *y*-dimensions for vertical and horizontal bars. We simulated a visual display (i.e., monitor) by creating a rectangular area assigned a luminance value of 0, while the simulated offscreen area was assigned a luminance value of −1. Bar stimuli were assigned a luminance value of 1. The display area ranged from −30 to 0°, while the offscreen area ranged from 0 to 30° along the *x*-dimension. Display edges were treated as either a mask or a boundary. When the display edge served as a mask, the bar stimuli incrementally moved off the display, disappearing behind the edge. However, when the display edge served as a boundary, the bar stimuli disappeared when the leading edge met the display edge. To show differences in orientation selectivity between masks and boundaries (Fig. 6*c*,*d*), we examined the response of an RF placed at the center of the FOV (0°, 0°). When simulating a grid of RFs (Fig. 6*e*), the display area was changed to span −15 to 15° in the *x*- and *y*-dimensions to capture responses of RFs that extended beyond the display area (RFs spanned −20 to 20° in *x*- and *y*-dimensions), and the display edges were treated as boundaries (as there was no orientation selectivity for mask edges).

#### RF properties simulation

Circular apertures had a radius of 30°. Horizontal and vertical edge aperture conditions were made by masking either the bottom or left half of the circular aperture. Spatial frequency of the gratings *v_g_* was kept at 0.04 cycles/° (Fig. 3*a*, top row, center). For the investigation of preference-switching and preference-maintaining neurons (Fig. 8), 36 evenly spaced stimulus orientations were used.

### RF model responses

#### RF responses in visual coordinates (Fig. 1c)

The scalar response to each stimulus from each RF was calculated by performing a dot product between the vectorized images of each RF and stimulus. Responses were then normalized between 0 and 1 across all RF locations. This normalized response simulated the change in response from baseline driven by the presented visual stimuli. Independent Gaussian noise (mean: 0, standard deviation: 0.01) was then added to each normalized response. The total simulated response was calculated as follows:

$$R_{t}=\alpha R_{\Delta }+\beta +\omega ;\;\omega ∼N(0,\;0.01),\;(7)$$

where *R_t_* denotes the total response; *R*_Δ_ denotes the normalized, stimulus-driven change in response; *β* denotes a baseline response; *ω* denotes the Gaussian distributed noise; and *α* denotes a scaling factor on the stimulus-driven change in response. Throughout this study, the baseline response *β* was always set to have a value of 1, and *α* was also always set to have a value of 1. This resulted in a maximum possible increase (before adding noise) from baseline response of 100% (*α/β* = 1). If a different maximum increase were desired, the ratio of *α* to *β* could be changed correspondingly (e.g., *α/β* = 0.75 for a 75% maximum increase).

The matrix of responses for the set of center-surround RFs was a 4D matrix of size 12 (stimulus orientations) × 4 (stimulus phases) × {58,081, 103,041, 160,801} (RF locations for either ±30°, ±40°, or ±50° RF grids) × 2 (RF phases), while the matrix of V1-like RF responses had an additional fifth dimension of size 8 for each RF orientation. For center-surround RFs, responses across RF phases were collapsed by thresholding negative responses to 0 and summing the On-center and Off-center responses together. For V1-like RFs, responses across RF phases were collapsed by squaring quadrature phase responses and summing the two together, which is the formalized method to achieve phase-invariant energy responses for Gabor RFs (Adelson and Bergen, 1985). Collapsing responses across RF phases resulted in a matrix of size 12 × 4 × {58,081, 103,041, 160,801} for center-surround RFs or 12 × 4 × {58,081, 103,041, 160,801} × 8 for V1-like RFs. Responses were then averaged across stimulus phases, resulting in a matrix of size of either 12 × {58,081, 103,041, 160,801} for center-surround RFs or 12 × {58,081, 103,041, 160,801} × 8 for V1-like RFs.

For the bar stimulus simulations, a baseline response was calculated from periods in which no bar was displayed. Determining this baseline was necessary because a model RF along a black/gray border, i.e., simulated display edge, produces a nonzero response even without a bar being presented (as opposed to zero for a purely gray background). This baseline response was then subtracted from the response to each bar presentation, resulting in a response of 0 when no bar overlapped with the RF. RF responses were normalized to a maximum response of 1 across all possible presentations of horizontal and vertical bar stimuli. The mean response across all horizontal bars was taken as the horizontal response, and the mean response across all vertical bars was taken as the vertical response. Orientation preference was determined by the higher mean response of vertical vs horizontal bar orientations.

#### RF responses in anatomical coordinates (Fig. 1d)

To compare model results with measurements from Liang et al. (2023), we developed a method for simulating model responses in anatomical coordinates. RF responses were approximated at anatomical locations using the previously simulated RF responses in visual coordinates. This process can be summarized in two steps: (1) defining a set of corresponding anatomical and visual coordinates and (2) generating responses for those coordinates.

**Figure 2.**
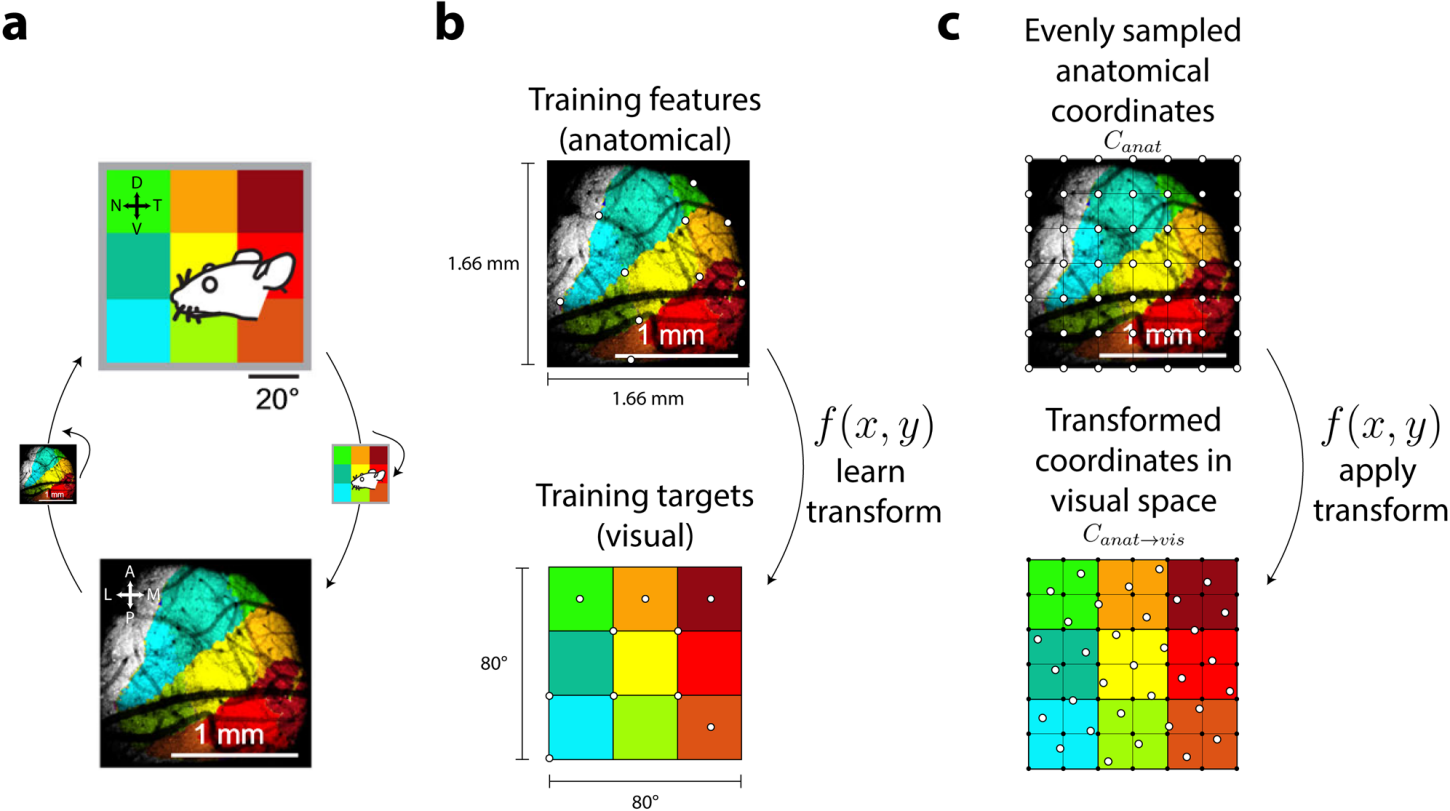
Aligning anatomical and visual coordinates. ***a***, Map of visual space (top) and corresponding aligned coordinates in anatomical space (bottom) for an example mouse [adapted from Liang et al. (2023), their Fig. 1]. ***b***, Mapping anatomical space (top) to visual space (bottom) requires transforming anatomical input coordinates to visual output coordinates. Functions approximating these transforms through natural neighbor interpolation were learned using a set of defined training features (top, white points) and a set of training targets (bottom, white points). ***c***, Once learned, functional transforms were applied to convert given points in anatomical space (top, white points) to an approximate location in visual space (bottom, white points). RF responses at these locations were estimated as weighted averages of previously simulated RF responses in visual space (bottom, black points).

To define anatomical coordinates, we generated an evenly spaced grid of coordinates in anatomical space. Natural neighbor interpolation (Sibson, 1981) was used to learn an approximate transform from anatomical to visual space coordinates (see following paragraph for brief overview). This was implemented by training two nonlinear interpolant functions that estimated *x*- or *y*-coordinates in visual space given pairs of *x*–*y* coordinates in anatomical space. Using the retinotopic maps provided by Liang et al. (2023) (Fig. 2*a*), we trained these two functions by designating 10 points in anatomical space as training features and the 10 corresponding points in visual space as training targets (Fig. 2*b*, white points). The extent of the square anatomical window (Fig. 2*a*, bottom) was defined as ±0.83 mm, and the extent of the square visual field grid (Fig. 2*a*, top) was defined as ±40°, following the scale illustrated in the retinotopy. We defined an evenly spaced grid (241 × 241 samples) of anatomical coordinates, *C*_anat_ (Fig. 2*c*, top, white points), overlaying the anatomical map. The two nonlinear interpolant functions were then used to find visual coordinates, *C*_anat→vis_ (Fig. 2*c*, bottom, white points), corresponding to *C*_anat_.

In natural neighbor interpolation, given a set of training features in the input space (e.g., a set of *x*- and *y*-coordinates in anatomical space) and a set of training targets in the output space (e.g., a set of *x*-coordinates or a set of *y*-coordinates in visual space), a nonlinear function, based on Voronoi tessellation, is learned that transforms any feature in the input space to a corresponding target in the output space. As opposed to linear interpolation, the nonlinearity afforded by natural neighbor interpolation better approximated the nonlinear transform from anatomical to visual coordinates. The benefit of a more complex, nonlinear transform can be qualitatively appreciated when comparing anatomical to visual space (Fig. 2*a*, note distortion of the square grid in anatomical space).

To generate RF responses at coordinates *C*_anat→vis_, a linear weighting was performed on previously simulated RF responses (as described above, RF responses in visual coordinates). The response at any coordinate *C*_anat→vis_ was estimated as a weighted average of RF responses from the four closest surrounding coordinates from the previously simulated grid of RF responses (Fig. 2*c*, bottom panel; RF responses at the four black points surrounding the top-leftmost white point). The weighted contribution from each surrounding RF response was determined by the spatial distance to the coordinate *C*_anat→vis_. Any *C*_anat→vis_ coordinates falling outside the range of the previous RF response grid were excluded. For V1-like RFs, weighted responses at coordinates *C*_anat→vis_ were first calculated for each possible RF orientation (of 8), and afterward, the response from a random RF orientation was chosen at each coordinate location.

#### Population RF responses (Fig. 1e)

We used spatial smoothing to approximate population responses. A 2D Gaussian smoothing kernel was applied across responses simulated at anatomical coordinate locations *C*_anat_, with a standard deviation of 0.0083mm, or 0.44° in visual coordinates (on average, owing to a nonlinear transform between anatomical and visual space). In terms of RF coverage, responses from a grid of ∼16–25 RFs (4 × 4 to 5 × 5) would fall within 1 standard deviation of the smoothing kernel.

#### Single-unit orientation tuning (Fig. 8e)

Single-unit tuning functions were obtained by simulating responses of either a single center-surround or V1-like RF to 36 evenly spaced orientations from 0 to 180° and fitting the set of responses with a circular von Mises function, using the Circular Statistics Toolbox through Matlab (Berens, 2009). The curve was duplicated from 180 to 360° to visually match the tuning curves obtained from empirical direction selectivity measurements (Fig. 8*d*).

### Orientation preference maps and orientation selectivity index

Global orientation selectivity indices (gOSI) were calculated based on the following equation:

$$gOSI=\frac{\left|\Sigma_{\theta_{\mathrm{stim}}}R_{x,y}(\theta_{\mathrm{stim}})e^{i2\theta_{\mathrm{stim}}}\right|}{\Sigma_{\theta_{\mathrm{stim}}}|R_{x,y}(\theta_{\mathrm{stim}})|},\;(8)$$

where $\theta_{\mathrm{stim}}$ denotes stimulus orientation (in radians) and *R* denotes the matrix of responses across RF locations. We normalized gOSI from 0 to 1 across the set of responses from each RF across all stimulus orientations for each stimulus manipulation (i.e., size, shape, spatial frequency). We used gOSI for both thresholding and as alpha transparency values in visualizations of orientation preference maps to highlight RFs based on their degree of orientation selectivity.

Orientation selectivity for bar stimuli was calculated based on the following equation:

$$OS_{\mathrm{bar}}=\frac{\left|{\bar{R}}_{h(x,y)}\;-\;{\bar{R}}_{v(x,y)}\right|}{\left|{\bar{R}}_{h(x,y)}|\;+\;|{\bar{R}}_{v(x,y)}\right|},\;(9)$$

where ${\bar{R}}_{h(x,y)}$ denotes the mean response of the RF centered at (*x*, *y*) across all horizontal bars, ${\bar{R}}_{v(x,y)}$ denotes the mean response of the RF centered at (*x*, *y*) across all vertical bars, and OS_bar_ denotes the orientation selectivity of the RF, ranging from 0–1. Orientation preference was determined based on the values of ${\bar{R}}_{h(x,y)}$ and ${\bar{R}}_{v(x,y)}$. A larger value of ${\bar{R}}_{h(x,y)}$ over ${\bar{R}}_{v(x,y)}$ was taken as preference for horizontal orientations, while a larger value of ${\bar{R}}_{v(x,y)}$ over ${\bar{R}}_{h(x,y)}$ was taken as preference for vertical orientations. OS_bar_ was used as alpha transparency values for visualization, indicating degree of selectivity in orientation preference maps to bar stimuli.

## Results

### Center-surround RFs with fixed properties produce radial orientation preferences

To determine what aspects of the imaging results can be accounted for by RFs not intrinsically selective for stimulus orientation, we built a computational model using only circular-symmetric, center-surround RFs. RFs tiled the visual field with size (*σ*_center_, 1.5°; *σ*_surround_, 11.4°) and peak spatial frequency preference (0.04 cycles/°) aligned with previously reported measures of RF parameters in mouse sSC (Wang et al., 2010; Inayat et al., 2015; Kasai and Isa, 2016; Ito et al., 2017; De Franceschi and Solomon, 2018). Stimuli were oriented gratings with parameters for orientation, spatial frequency, aperture shape, and size matching those used by Liang et al. (2023) (Fig. 1*b*). Model single-unit RF responses were obtained by computing the dot product between the stimuli and RFs, followed by thresholding and summing resulting values across pairs of On-center, Off-surround and Off-center, On-surround RFs (Fig. 1*c*).

To compare the results of the model to wide-field Ca^2+^ imaging data, we spatially transformed the simulated responses to better match the anatomical coordinates in mouse sSC (Fig. 1*d*). Using maps of visual and anatomical space provided in the Liang et al. (2023) study (Fig. 2*a*), we constructed a set of functions that estimated visual coordinate locations from anatomical coordinate locations (Fig. 2*b*). This transformation resulted in a roughly 90° clockwise rotation and spatial warping from visual to anatomical space (Fig. 2*a*, inset transformation, right). Single-unit RF responses at a simulated grid of anatomical coordinates were then estimated by transforming RF responses for corresponding coordinates in visual space (Fig. 2*c*). Each pixel in the Ca^2+^ imaging measurement reflected the joint activity of a population of neurons. Hence, to simulate these measurements, we spatially smoothed responses from the simulated RFs in anatomical coordinates using a Gaussian filter with a standard deviation of 8.3 microns (∼0.44° of visual space).

**Figure 3.**
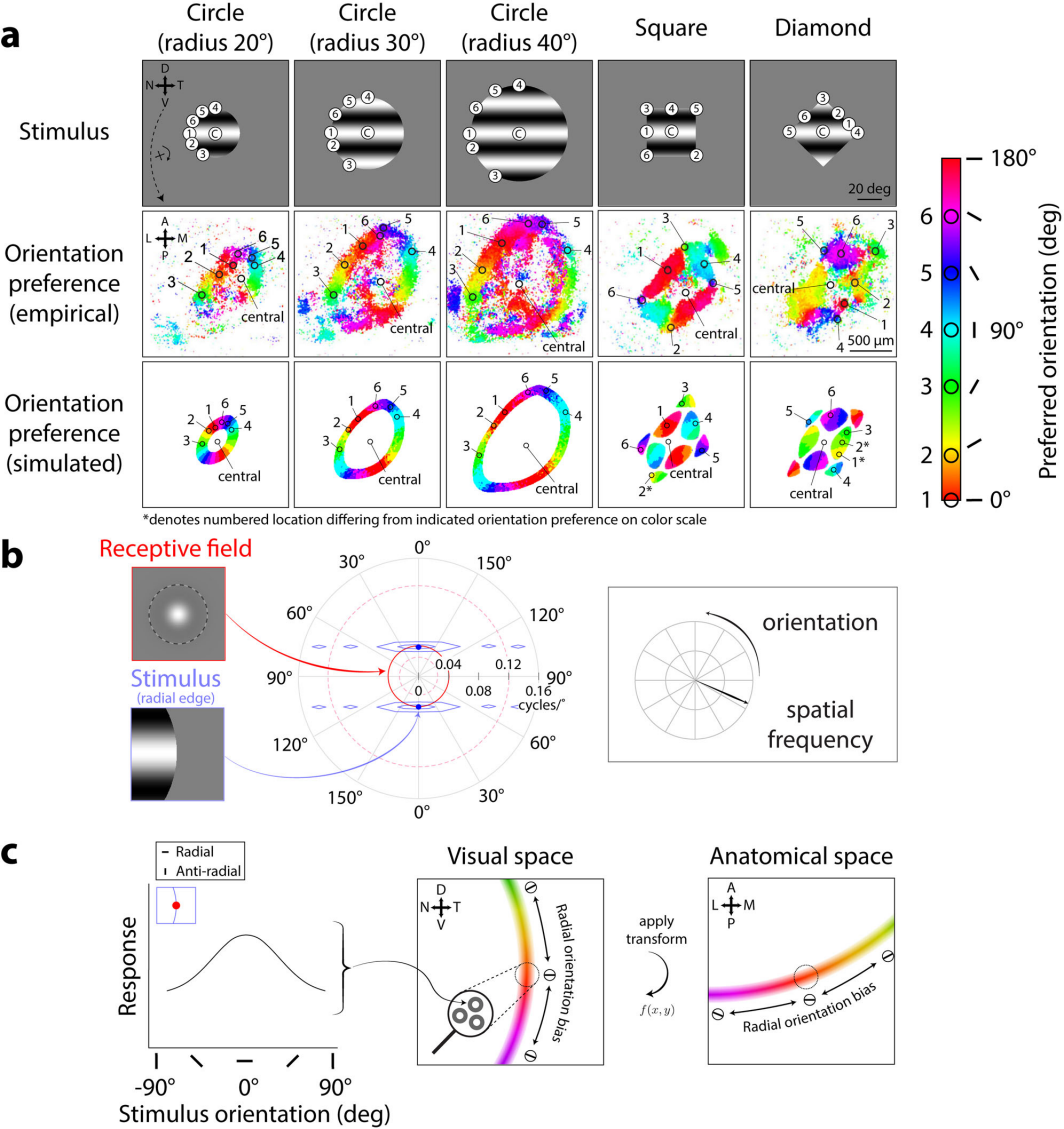
Center-surround RF model predicts orientation preference map changing across aperture size and shape. ***a***, Orientation preference maps from Ca^2+^ imaging [middle row; adapted from Liang et al. (2023), their Fig. 2] and RF model simulations (bottom row) to oriented grating stimuli of varying aperture shapes and sizes (top row). Map colors correspond to preferred orientation, see scale. Numbered circles in color scale and in the middle row, leftmost panel were defined by Liang et al. (2023) to show varied orientation preferences around SC; we matched these numbered circles onto all other panels to illustrate matching retinotopic and anatomical locations. “C” (top row): central. ***b***, Idealized interaction in spatial frequency domain between center-surround RF (red inset) and an edge that is perfectly vertical (blue inset). Overlap between RF (red solid/dashed circles, center) and stimulus (blue envelopes, center) determines RF response magnitude. Solid red circle denotes RF center (preferred) spatial frequency; dashed red circles denote ±1 standard deviation from center spatial frequency in log-units. ***c***, Left panel, Model single-unit center-surround RF responses across stimulus orientations at vertical aperture edge (inset). Center and right panels: Population RF orientation preferences (oriented markers and color gradient, same color scale as in ***a***) from averaging single-unit RF responses across spatial locations (e.g., center panel, zoom-in), shown in visual (center) and corresponding anatomical coordinates (right).

Simulated RF responses for stimuli with different apertures resulted in qualitatively similar aperture-dependent orientation preferences as seen in the mouse sSC imaging data. For example, consider RF orientation preferences for the smallest circular aperture (Fig. 3*a*, 20° radius column). In the model and the mouse sSC data, RFs responsive to locations along the stimulus aperture (Fig. 3*a*, radius 20°, top panel, locations 1–6) demonstrated robust orientation preferences (Fig. 3*a*, compare middle and bottom panels, locations 1–6). In contrast, RFs responsive to locations away from the aperture, such as toward the center of the stimulus (Fig. 3*a*, radius 20°, top panel, location “central”), tended not to exhibit orientation preferences (Fig. 3*a*, compare middle and bottom panels, location “central”). Notably, the orientation preferences from both the imaging data and model predictions varied systematically along the aperture: the preferred orientation at each RF location was orthogonal to the aperture edge. We refer to this phenomenon as a preference for radial orientations. While this term is sometimes used to denote orientations radial with respect to the fovea (Sasaki et al., 2006; Mannion et al., 2010; Freeman et al., 2011), here we use it to describe orientations radial to the center of the stimulus. For example, an RF responsive to the horizontal (top) edge of the aperture (Fig. 3*a*, radius 20°, top panel, location 4) preferred a vertical orientation (Fig. 3*a*, compare middle and bottom panels, location 4, see also Fig. 3*a* color bar, circle 4, cyan), while an RF responsive to the vertical (left) edge of the aperture (Fig. 3*a*, radius 20°, top panel, location 1) preferred a horizontal orientation (Fig. 3*a*, compare middle and bottom panels, location 1; see also Fig. 3*a* color bar, circle 1, red). Likewise, RFs responding to other locations along the aperture (Fig. 3*a*, radius 20°, top panel, locations 2, 3, 5, 6) also demonstrated orientation preferences orthogonal to their respective aperture edges (Fig. 3*a*, compare middle and bottom panels, locations 2, 3, 5, 6; orientation preferences: 15°, 60°, 120°, 150°, see color bar circles 2, 3, 5, 6).

Across aperture sizes, orientation preference maps predicted by the model provided a qualitative match to the maps from imaging data. In response to larger circular apertures (Fig. 3*a*, radius 30° and 40°, top panels), the circular pattern of RF orientation preferences expanded in both the simulated results and the imaging data. This expansion also resulted in a larger retinotopic region corresponding to the center of the stimulus (Fig. 3*a*, radius 30° and 40°, top panels, locations around central) lacking orientation preferences (Fig. 3*a*, radius 30° and 40°, middle and bottom panels, non-colored regions around “central”). Although the locations in the sSC exhibiting orientation preference changed with the size of the aperture, radial orientation preferences remained consistent (Fig. 3*a*, compare orientation preference color gradients, i.e., red → orange → yellow → …, across radius 20°, 30°, 40°, middle and bottom panels).

Across aperture shapes, simulated orientation preference maps also qualitatively matched the maps from imaging data. For the square and diamond apertures (Fig. 3*a*, square and diamond columns, top panels), the previously demonstrated circular patterns of RF orientation preferences now adopted rectangular patterns, shown in both the model and imaging data (Fig. 3*a*, square and diamond columns, compare middle and bottom panels). RFs lacked orientation preferences at locations near the aperture center (Fig. 3*a*, square and diamond columns, top row, locations around central). Along the straight edges of the square, the imaging data demonstrated clear radial orientation preferences (Fig. 3*a*, square column, middle panel, location 1, orientation preference: 0°, red; location 4, orientation preference: 90°, cyan). This pattern was also evident in the simulation (Fig. 3*a*, square column, bottom panel, location 1, orientation preference: 0°, red; location 4, orientation preference: 90°, cyan). At corner locations, the model again predicted orientation preferences similar to those measured in the imaging data (Fig. 3*a*, compare middle and bottom panels, square column, locations 2, 3, 5, 6; diamond column, locations 3, 4, 5). However, we note some difference in the orientation preference maps in response to the diamond aperture; the imaging data showed a more scattered set of orientation preferences along the aperture edge than the model (Fig. 3*a*, diamond column, compare orientation preferences of locations 1 and 2 between middle and bottom panels).

**Figure 4.**
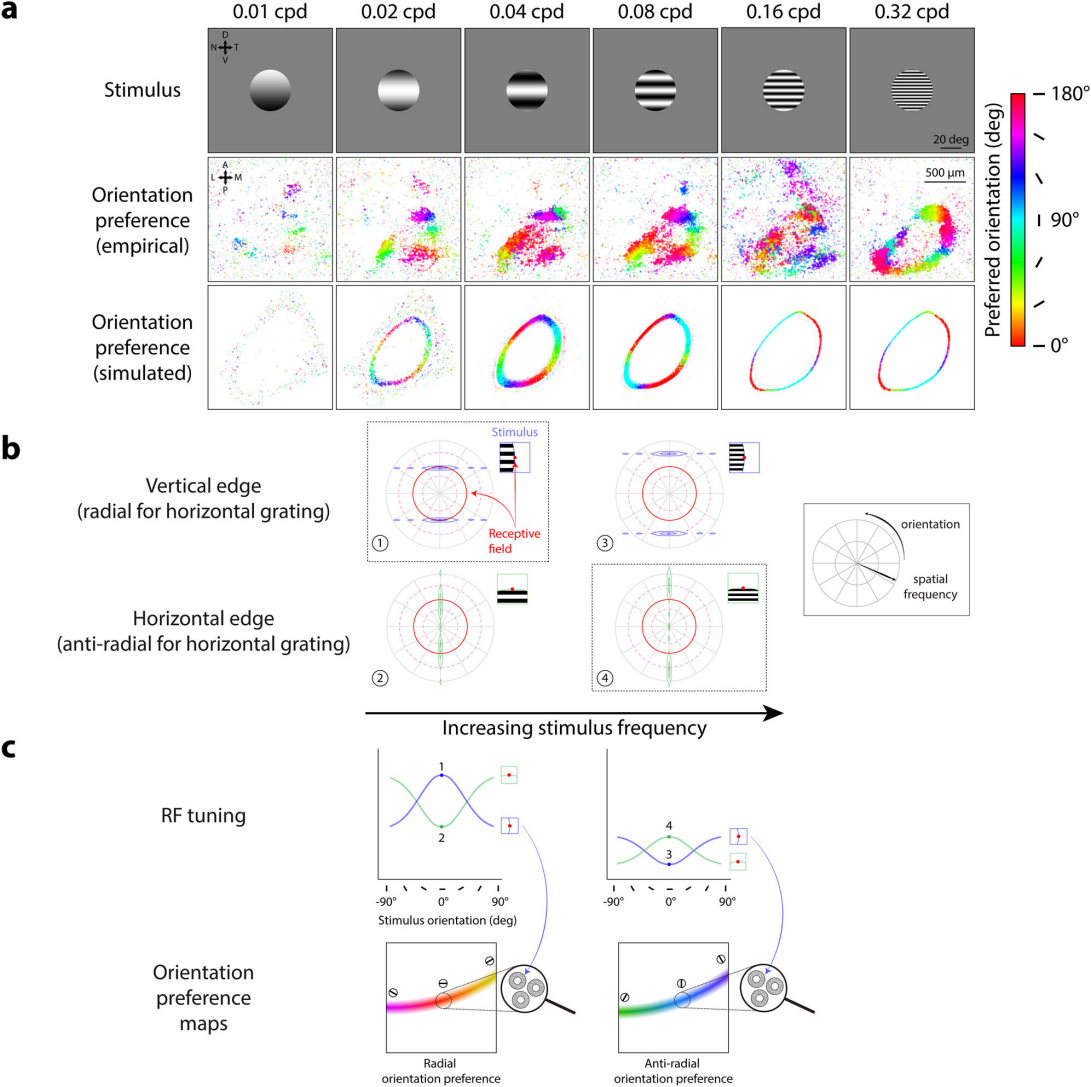
Center-surround RF model predicts orthogonal orientation preferences changing with stimulus spatial frequency. ***a***, Orientation preference maps from imaging data [middle row, adapted from Liang et al. (2023), their Supplementary Fig. 3] and model simulations (bottom row) in response to oriented grating stimuli of varying spatial frequencies; map colors correspond to preferred orientation, see scale; cpd, cycles/°. ***b***, Model prediction of largest RF responses (dotted black outlines) depending on overlap between RF (red) and horizontal stimulus grating at vertical (blue, panels 1, 3) or horizontal (green, panels 2, 4) edges. Left to right, Increasing stimulus frequency. Solid red circles denote RF center (preferred) spatial frequency; dashed red circles denote ±1 standard deviation from center spatial frequency. ***c***, Model prediction of orientation preferences across stimulus spatial frequencies; left to right, low to high spatial frequency. Top row, Orientation tuning curves for single-unit RFs at vertical (blue, see inset) and horizontal aperture edges (green, see inset); numbered points correspond to panels in ***b*** (RF responses to horizontal gratings). Bottom row, Orientation preference maps for population RFs across simulated anatomical locations. Dotted circles show example population RFs around the vertical aperture edge, generated by averaging local single-unit RF responses with tuning profiles shown in the top row (blue arrows); color gradient (see scale in ***a***) and oriented markers indicate varying population orientation preferences along the stimulus aperture.

The reason the center-surround RF model produced radial orientation preferences can be readily appreciated by considering the overlap of the stimulus energy with RFs. We first provide a heuristic explanation in the spatial domain. At any edge of a grating stimulus, the stimulus can be thought of as having two different orientations—the orientation of the grating and the orientation of the edge. For example, at the rightmost edge of the stimulus, a sinusoidal grating vignetted by an aperture contains both the horizontal orientation of the grating, as well as the vertical orientation of the aperture edge (Fig. 3*b*, blue inset). For a circular-symmetric RF not intrinsically selective for orientation (Fig. 3*b*, red inset), radial orientations, which primarily have stimulus energy at these two orthogonal orientations, produce a larger RF response compared with “anti-radial” orientations (i.e., stimulus orientations running parallel to edge orientation; see Fig. 4*b*, bottom row insets for examples) where there is only a single orientation in the stimulus [see Gardner and Merriam (2021) for an in-depth discussion of this phenomenon].

The explanation for radial orientation preferences is more apparent by considering the RF and stimulus in the spatial frequency domain. Because the model center-surround RFs were constructed as a difference-of-Gaussians in the spatial domain, the RFs in the spatial frequency domain were also difference-of-Gaussians (Enroth-Cugell and Robson, 1966). Thus, these RFs formed a donut-like shape in the spatial frequency domain (Fig. 3*b*, red solid/dashed circles) selective for spatial frequency. To determine how such a linear RF would respond to a stimulus, we can examine the overlap of the stimulus with the RF in the spatial frequency domain. The response is the pointwise product of the RF and the stimulus, summed across all spatial frequencies. For example, consider such an RF with a center spatial frequency of 0.04 cycles/° (Fig. 3*b*, red inset) that overlaps with the rightmost vertical, i.e., radial, edge of a horizontally oriented sinusoidal grating, also with a spatial frequency of 0.04 cycles/° (Fig. 3*b*, blue inset). An infinitely large grating in the spatial domain is represented by two infinitesimally small points (Fig. 3*b*, blue points) in the spatial frequency domain, with their distance from the origin determined by the grating's spatial frequency (e.g., 0.04 cycles/°). For a horizontal grating (0°/180° orientation), the angular locations correspond to 90 and 270° in polar angle. The presence of an aperture edge spreads the stimulus energy across orientations and spatial frequencies, depending on the orientation of the edge. For example, a perfectly vertical aperture edge spreads stimulus energy horizontally, primarily across many orientations, in the spatial frequency domain (Fig. 3*b*, center, blue envelope). To generalize, any oriented edge will spread stimulus energy in the orthogonal direction in the spatial frequency domain (e.g., a horizontal aperture edge would spread energy vertically). Critically, this means that the radial aperture edge will spread stimulus energy primarily across many orientations. In the case that the stimulus spatial frequency is near the RF's center spatial frequency, the stimulus orientation creating a radial aperture edge (in this example, a vertical aperture edge for a horizontal grating stimulus) creates the largest overlap between stimulus and RF (Fig. 3*b*, blue and red overlap), compared with any other orientation. Therefore, grating orientations that create a radial aperture edge are predicted to generate the largest RF response out of all possible orientations (Fig. 3*c*, left panel).

Extending this logic to a spatially distributed population of center-surround RFs, we arrive at the model predictions described earlier (Fig. 3*a*, bottom row). Orientation preference maps changed dynamically based on stimulus shape and size in the imaging data (Fig. 3*a*, middle row). The model shows that preferences for radial orientations systematically explain this phenomenon because a spatial distribution of single-unit center-surround RFs preferring radial orientations should produce a gradient of radial orientation preferences varying smoothly along the aperture edge. This can be appreciated intuitively in visual coordinates (Fig. 3*c*, center) and equivalently in anatomical coordinates (Fig. 3*c*, right) for comparison to the imaging data. Additionally, anatomical locations representing the center of the stimulus tended to lack orientation preferences (Fig. 3*a*, middle row, non-colored locations around “central”). This too is predicted by the model, because where there is no spreading of orientation energy (i.e., where RFs do not overlap the stimulus edge), there should be no preferred orientation. Across stimulus shapes and sizes, the logic of stimulus and RF overlap implemented by the model captures the stimulus-dependent nature of orientation preferences found in the imaging data. In summary, linear circular-symmetric RFs are sufficient to account for the stimulus-dependent dynamic orientation phenomena observed in prior imaging data.

### Orientation preference maps depend on stimulus spatial frequency

**Figure 5.**
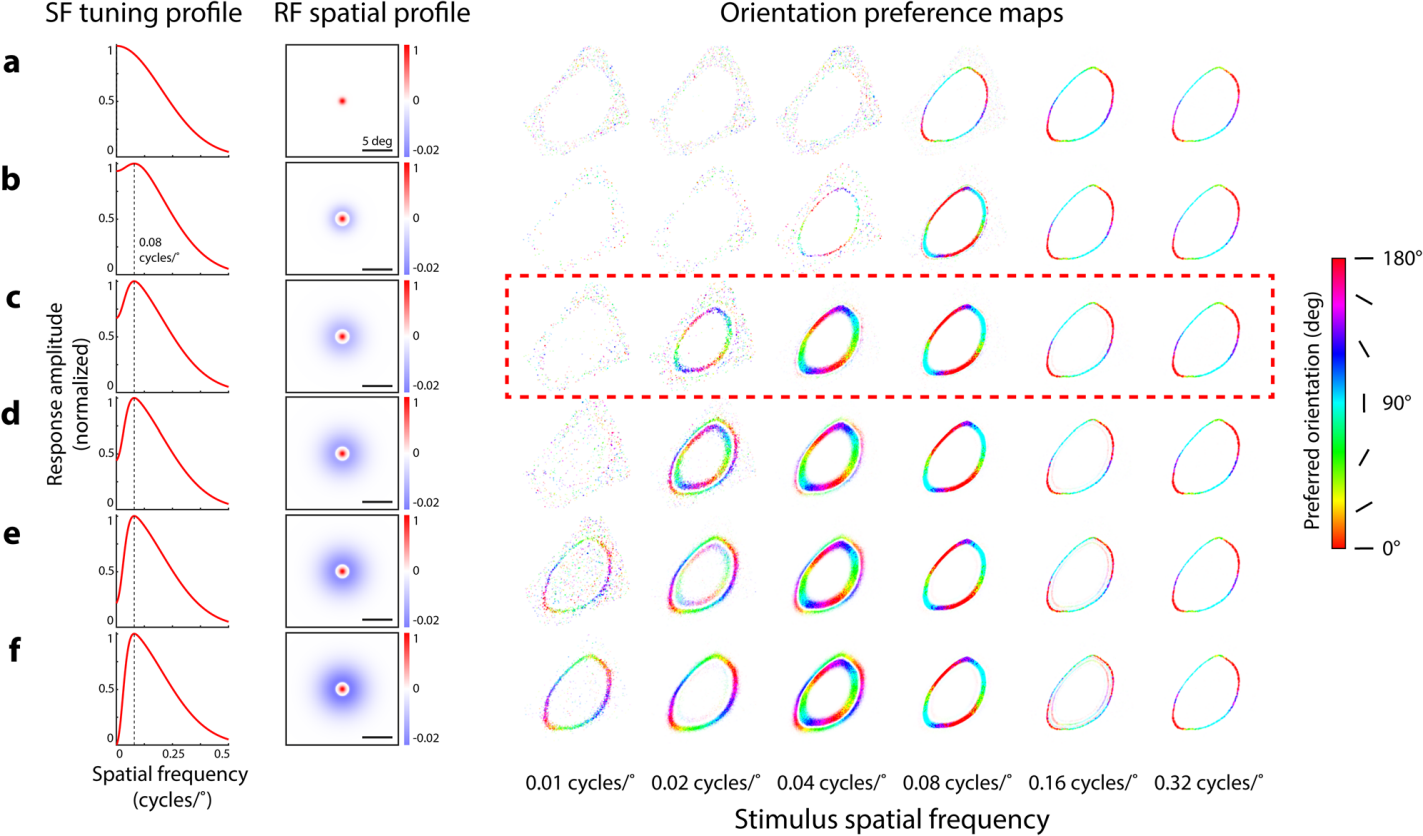
Impact of surround size and amplitude on simulated orientation preference maps. ***a***, Simulated orientation preference maps (right) produced by spatially distributed, circular-symmetric Gaussian RFs (center size, *σ*: 0.8°) in response to circular grating stimuli with a radius of 30°. Colors correspond to orientation preference (color scale, right). ***b–f***, Simulated orientation preference maps as described in ***a***, using center-surround RFs with varying center-surround size (*σ*) ratios and center-surround amplitude ratios to maintain a preferred spatial frequency (SF) of 0.08 cycles/° (dashed vertical lines, left column) across all RF manipulations. Dashed red box corresponds to the same orientation preference maps shown in Figure 4. Center to surround size ratios (***b–f***): 0.8° to 3, 4.7, 5.2, 5.5, 5.7°; center to surround amplitude ratios: 1 to 0.2, 0.4, 0.6, 0.8, 1. Responses in SF tuning profiles normalized from 0 to 1, after thresholding and summing responses from pairs of opposite polarity center-surround RFs (see Materials and Methods). Amplitudes of RF spatial profiles were normalized between possible values of −1 to 1 (scale shown from −0.02 to 1 for clearer visualization of On-center RFs, paired Off-center RFs not shown). Amplitude ratio refers to the ratio of volumes under the circular 2D center and surround Gaussians used to construct the difference-of-Gaussians center-surround RFs.

The model predicts that radial orientation preferences should change with the spatial frequency of the stimulus, an effect that was also found in mouse sSC data. Orientation preferences in mouse sSC were previously measured (Liang et al., 2023) using a set of stimuli varying in spatial frequency (Fig. 4*a*, top row), spanning spatial frequency preferences reported in the mouse sSC literature (Wang et al., 2010; De Franceschi and Solomon, 2018). The imaging results showed that the most robust representation of radial orientation preferences appeared for stimuli with spatial frequencies of 0.04 and 0.08 cycles/° (Fig. 4*a*, 0.04 and 0.08 cycles/° column, middle row). To simulate RFs that could reproduce this phenomenon, we performed a grid search across a range of center-surround RF parameters (center-surround size and amplitude ratios, which vary RF spatial frequency tuning) to find RF parameters producing orientation preference maps that best matched the imaging results qualitatively (see Fig. 5 and accompanying text for detail). Center-surround RFs with a size (σ) ratio of 0.8° to 4.7° and surround-to-center amplitude ratio of 0.4 to 1 (resulting in a preferred spatial frequency of 0.08 cycles/°) predicted orientation preferences (Fig. 4*a*, 0.04, 0.08 cycles/° columns, bottom row) best matching the empirical observations (Fig. 4*a*, 0.04, 0.08 cycles/° columns, middle row), demonstrating radial orientation preferences for stimuli with spatial frequencies of 0.04 and 0.08 cycles/°. At lower stimulus spatial frequencies, the imaging data demonstrated a fading of the radial orientation preference map (Fig. 4*a*, 0.01, 0.02 cycles/° columns, middle row), which is also predicted by the model (Fig. 4*a*, 0.01, 0.02 cycles/° columns, bottom row).

Notably, the results from Liang et al. (2023) revealed that at high stimulus spatial frequencies, orientation preferences shifted from radial orientation preferences to anti-radial orientation preferences, a result that the center-surround RF model also captured. We refer to anti-radial orientations as orientations parallel to the edge, i.e., orthogonal to radial orientations. anti-radial orientation preferences were evident for the highest stimulus spatial frequency in both the imaging data (Fig. 4*a*, middle row, 0.32 cycles/°) and the model-predicted orientation preference maps (Fig. 4*a*, bottom row, 0.32 cycles/°).

The emergence of anti-radial rather than radial orientation preferences can be explained by the model when considering the spatial frequency representations of stimuli with frequencies higher than the RF's preferred spatial frequency (Fig. 4*b*, right column). For example, consider a horizontal stimulus grating (Fig. 4*b*, insets). As stimulus spatial frequency is increased, stimulus energy in the spatial frequency domain is shifted outward, meaning that stimuli with higher spatial frequency are now pushed toward the outer edge of the RF (Fig. 4*b*, compare stimulus energy locations between left and right columns). A vertical (radial) edge spreads stimulus energy across a much smaller portion of the RF at high spatial frequencies (Fig. 4*b*, panel 3), compared with when stimulus and RF preferred spatial frequencies are matched (Fig. 4*b*, panel 1). In contrast, the effect of increasing spatial frequency is less consequential when considering stimulus-RF overlap for a horizontal (anti-radial) edge because stimulus energy is spread toward the center of the spatial frequency domain (Fig. 4*b*, bottom row, compare stimulus-RF overlap between panels 2 and 4). Because the anti-radial aperture edge now spreads a larger amount of stimulus energy across the RF than the radial aperture edge (Fig. 4*b*, right column, compare stimulus-RF overlap between panels 3 and 4), we observe a larger RF response at the anti-radial over the radial aperture edge (Fig. 4*c*, right column, top panel, compare responses at points 3 and 4). Averaging across responses from local single-unit RFs, the model predicts a gradient of anti-radial orientation preferences across the population along the aperture edge (Fig. 4*c*, right column, bottom panel).

A divergence between the model and imaging data in the transition from radial to anti-radial orientation preferences highlights how the model RFs might differ from populations in mouse SC. For the 0.16 cycles/° stimulus, the imaging data appeared to be undergoing a somewhat messy, non-uniform transition between radial and anti-radial orientation preferences (Fig. 4*a*, 0.16 cycles/° column, middle panels), but the model produced a clear map of anti-radial orientation preferences. The difference between the two could be due to several factors. Since the model predicted a transition between 0.08 and 0.16 cycles/°, the modeled population RFs’ spatial frequency tuning profile may not have exactly matched those in mouse sSC. Additionally, the model's simplified assumption of uniform RF properties across mouse sSC may have played a role as well. Population RFs pooled from many single units with a range of different RF properties may better recapitulate the less uniform transition from radial to anti-radial orientation preferences across stimulus spatial frequencies.

### Changes in orientation preference maps depend on RF parameters

Simulations of different center-surround RF properties revealed that small changes in RF properties can alter the transition between radial and anti-radial orientation preference maps. In a grid search procedure, we generated orientation preference maps to stimulus spatial frequencies ranging from 0.01 to 0.32 cycles/° using RFs varying in center-surround size and amplitude ratios while constrained to a particular preferred spatial frequency (Fig. 5*a–f*, SF tuning profile, RF spatial profile columns). As surround amplitude increased, we found the emergence of anti-radial orientation preferences at low spatial frequencies (Fig. 5*e*,*f*, orientation preference maps, 0.01 and 0.02 cycles/° columns). Higher surround amplitudes also resulted in orientation maps with inner and outer rings of opposing orientation preferences (Fig. 5*d–f*, orientation preference maps, 0.02 and 0.04 cycles/° columns). Notably, model RFs without a surround were unable to produce maps showing radial orientation preferences (Fig. 5*a*, orientation preference maps).

While small differences in RF properties changed where the radial to anti-radial transition point occurred, all modeled center-surround RFs showed a transition to anti-radial orientation preferences at higher stimulus spatial frequencies, similar to the imaging results from mouse SC. We found that maps of anti-radial orientation preferences produced in response to high spatial frequency stimuli were robust across our manipulations of RF parameters (Fig. 5*a–f*, orientation preference maps, 0.16 and 0.32 cycles/° columns). Similar patterns of results were found for RFs constrained to other preferred spatial frequencies (0.02, 0.06, 0.1 cycles/°, data not shown). The only notable difference caused by a change in preferred spatial frequency was the shifting of stimulus spatial frequency where the transition from radial to anti-radial orientation preferences was observed. Altogether, we found that orientation preference maps were sensitive to manipulations of surround amplitude and size, even while maintaining the same preferred spatial frequency. Our results suggest that the overall shape of the spatial frequency tuning curve, not just the peak tuning, dictates how patterns of orientation preferences manifest in sSC populations.

### Orientation preferences in center-surround RFs from oriented bar stimuli

**Figure 6.**
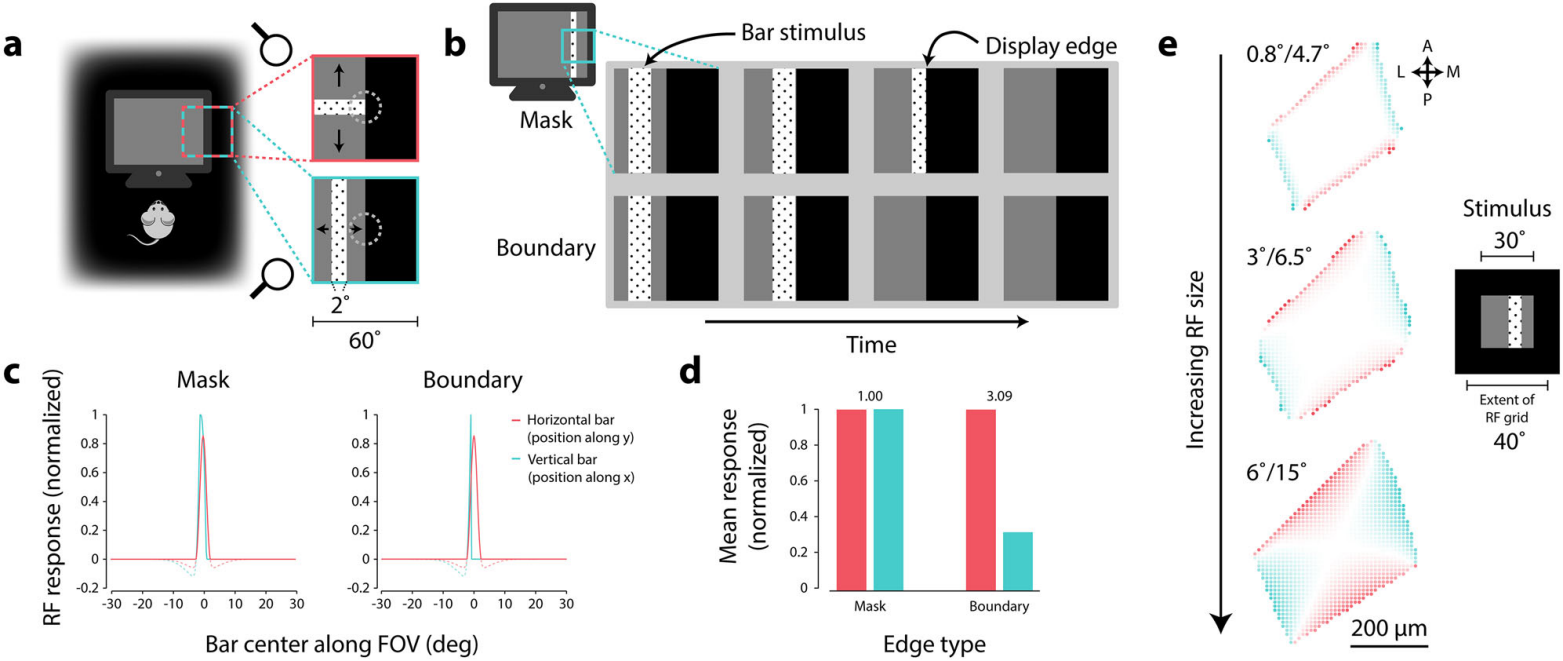
Bar stimuli can produce orientation preferences along display edges. ***a***, Simulation of circular-symmetric, center-surround RF (center size: 0.8°, surround size: 4.7°, surround amplitude: 0.4) located along the vertical edge of display apparatus, viewing either a horizontal (red) or vertical (cyan) bar stimulus. ***b***, Display edges simulated as either a mask (top row) or boundary (bottom row). ***c***, RF response along different bar locations for horizontal and vertical bars, using either a mask (left) or boundary (right) edge. Dashed plots show non-thresholded center-surround responses. ***d***, Differences in mean response to bar orientation depending on edge type. Values above bars indicate ratio of horizontal to vertical response. ***e***, Simulated bar orientation preferences across grid of RFs tiling anatomical space, across different RF sizes. Values at top left corner of each map indicate RF center/surround sizes. Inset (right) shows viewing area together with extent of RF grid in visual field coordinates. Screen edges (black-gray edges) were simulated as boundaries for bar stimuli. Color indicates preference for horizontal (red) or vertical (cyan) bars; transparency indicates degree of selectivity.

Simulations of center-surround RFs revealed orientation preferences not just for grating stimuli, but for bar stimuli as well. Along with oriented gratings, oriented bar stimuli are a common method to assess neural orientation selectivity (Hubel and Wiesel, 1962) and have also been used to demonstrate patterns of orientation selectivity in mouse sSC (Feinberg and Meister, 2015; Inayat et al., 2015; de Malmazet et al., 2018; Kasai and Isa, 2022; Li and Meister, 2023). Although drifting bar stimuli do not use a static aperture as with grating stimuli, we reasoned that edges of a visual display may still interact with bar stimuli to produce systematic, aperture-dependent orientation preferences in neural populations. We modeled RF responses along the edge of a simulated visual display edge in response to vertical and horizontal bar stimuli (Fig. 6*a*).

We found that the presence or absence of orientation selectivity depends on the details of how stimuli are displayed. When the display edge was treated as a mask that partially obscures the bar stimulus as it passes through the edge (Fig. 6*b*, top row), we found no difference in mean response between vertical and horizontal bars (Fig. 6*c*, left panel; Fig. 6*d*, left side). However, by treating the display edge as a hard boundary (i.e., stop presenting the bar when it reaches the display edge), there were substantial differences between mean responses to vertical and horizontal bars (Fig. 6*c*, right panel; Fig. 6*d*, right side). Simulating a grid of RFs extending beyond the simulated display area revealed that RFs along vertical display edges preferred horizontal bar stimuli, while RFs along horizontal display edges preferred vertical bar stimuli (Fig. 6*e*). Such preferences were consistent whether responses were linear or passed through a threshold nonlinearity and were consistent across a variety of RF sizes, though larger RFs showed a more widespread pattern of selectivity. These patterns were consistent with radial orientation preferences shown using oriented gratings.

The presence of orientation selectivity in the hard boundary scenario is due to differences in total time an edge RF is being stimulated by a vertical or horizontal bar. For example, consider a circular-symmetric RF along the vertical edge (such as the one shown in Fig. 6*a*). If vertical bars are stopped when the leading edge meets the vertical display edge (Fig. 6*b*, bottom row), the RF “sees” these bars for a shorter total period of time than corresponding horizontal bars moving up and down the display.

### RF structures from single-unit and population-scale measurements

**Figure 7.**
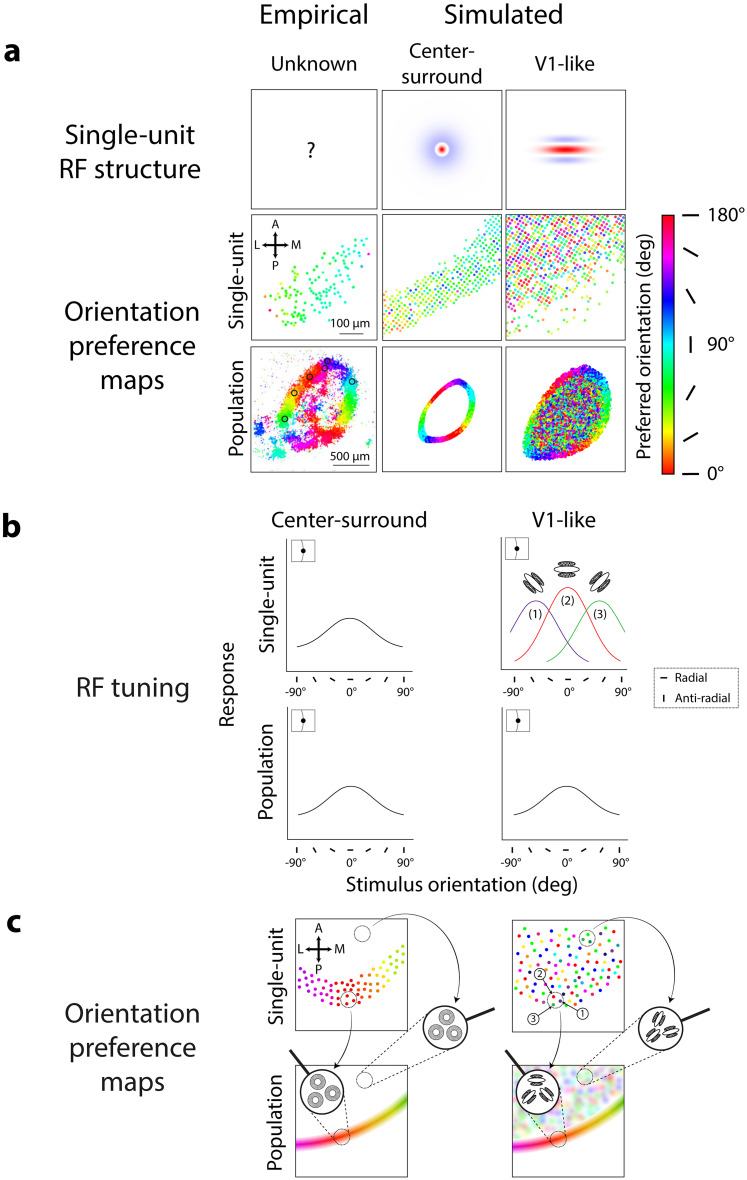
Center-surround and V1-like models produce different patterns of orientation preferences at population and single-unit scales. ***a***, Orientation preference maps from center-surround and V1-like RFs (top row) at single-unit (middle row) and population scales (bottom row) in response to circular grating stimuli (spatial frequency, 0.04 cycles/°; radius, 30°); colors correspond to orientation preference, see scale. Empirical maps (middle and bottom panels, left column) adapted from Liang et al. (2023), their Figures 2 and 3. ***b***, Model-predicted orientation tuning for center-surround and V1-like RFs at single-unit and population scale, shown for RFs located at the vertical aperture edge (insets). Color in top right panel corresponds to preferred orientation for example V1-like RFs 1–3 (see scale in ***a***). Legend denotes radial and anti-radial orientations for vertical aperture edges. ***c***, Model-predicted orientation preference maps for center-surround and V1-like RFs at single-unit and population scale for RFs distributed across SC. Responses from nearby single-units (top panels, dotted circles) are combined to generate local population orientation preferences (bottom panels, zoom-ins). Numbered units in top right panel correspond to example single V1-like units shown in ***b***; colors correspond to preferred orientation (see scale in ***a***).

The center-surround RF model not only behaved similarly to population data in mouse SC but also captured orientation preferences for single units measured with narrow-field imaging. Center-surround RFs in the model simulations produced single-unit orientation preferences (Fig. 7*a*, center column, middle panel) that qualitatively matched the single-unit imaging data (Fig. 7*a*, left column, middle panel), demonstrating radial orientation preferences dependent on edge orientation (Fig. 7*b*,*c*, left column, top panel). These simulated single-unit preferences were maintained at the population scale (Fig. 7*b*, left column, bottom panel), since the population response at each spatial location was simply obtained by averaging responses across nearby units (Fig. 7*c*, left column, bottom panel, dotted circle).

Intrinsically orientation selective V1-like RFs showed different orientation preferences at the single-unit scale compared with the imaging data and the center-surround RF simulations. Here, we define a V1-like RF as an RF with elongated subfields in parallel, as originally identified in V1 of cat and macaque (Hubel and Wiesel, 1959, 1968). We simulated V1-like RFs as oriented Gabor filters (Marĉelja, 1980; Pollen and Ronner, 1983; Jones and Palmer, 1987a,b; Gardner et al., 1999; Fig. 7*a*, top row, right column), with the same preferred spatial frequency as the simulated center-surround RFs (0.08 cycles/°) and with a random orientation at each spatial location. Simulated individual V1-like RFs thus had orientation preferences that varied randomly across model sSC (Fig. 7*a*, right column, middle panel), in contrast to the smooth gradient of orientation preferences in both the single-unit imaging data (Fig. 7*a*, left column, middle panel) and center-surround simulation (Fig. 7*a*, middle column, middle panel). Orientation preferences for simulated V1-like single units were scattered randomly because responses to different stimulus orientations depended largely on the intrinsically oriented RF structure (Fig. 7*b*, right column, top panel), making it so the stimulus aperture had minimal influence on orientation preferences (Fig. 7*c*, right column, top panel, example RFs 1–3 along stimulus edge).

V1-like RFs could, however, produce radial orientation preferences at the population scale. Simulated V1-like RFs at the population scale showed radial orientation preferences along the stimulus aperture (Fig. 7*a*, right column, bottom panel), as in the imaging data (Fig. 7*a*, left column, bottom panel) and the center-surround RF simulations (Fig. 7*a*, middle column, bottom panel). We note a larger overall region of orientation selective responses from V1-like RFs compared with center-surround RFs (Fig. 7*a*, bottom row, middle vs right panels) due to the larger size of the individual V1-like RFs. Radial orientation preferences for V1-like populations emerged because of the pooling of responses from randomly oriented V1-like single units. In theory, if each local population averages responses from units of all possible orientation preferences, the population RF will no longer be tuned for orientation and thus only be selective for spatial frequency. In practice, population RFs composed of many randomly oriented V1-like RFs closely approximate a circular-symmetric, center-surround RF. While the individual V1-like RFs themselves are largely immune to aperture-dependent orientation preferences (Fig. 7*a*, right column, middle row), the emergent center-surround-like population RF is quite susceptible to aperture-dependent radial orientation preferences (Carlson, 2014; Roth et al., 2018; Fig. 7*b*, right column, bottom panel), as with all RFs with circular-symmetric, center-surround structure. Therefore, along the stimulus aperture, population RFs composed of randomly oriented V1-like RFs demonstrate radial orientation preferences (Fig. 7*c*, right column, bottom panel, left zoom-in).

Without the influence of the stimulus aperture, these population RFs should ideally have no orientation preference, as with the center-surround RFs. However, the simulations demonstrated a patchy pattern of orientation preferences within the stimulus aperture (Fig. 7*a*, right column, bottom panel). This pattern appears because the simulations of population responses pool over V1-like units with varying orientation preferences, which may not be evenly distributed across all orientations at every possible location (Boynton, 2005; Kamitani and Tong, 2005; Fig. 7*c*, right column, top panel, zoom-ins). In cases where local anisotropies are present, pooling responses across these local regions can result in orientation preferences that vary randomly across the surface of simulated SC, though the heterogeneity of the local population generally results in weak orientation selectivity. In contrast, populations composed entirely of circular-symmetric, center-surround RFs do not show even weak selectivity (Fig. 7*c*, left column, top panel, right zoom-in), due to the individual units not having intrinsic orientation selectivity at all. Thus, population RFs composed of V1-like RFs can demonstrate intrinsic and scattered, though weakly selective, orientation preferences, determined by the preferences of their underlying single units (Fig. 7*c*, right column, bottom panel, right zoom-in).

### Preference-maintaining mouse sSC neurons are better modeled as V1-like RFs

**Figure 8.**
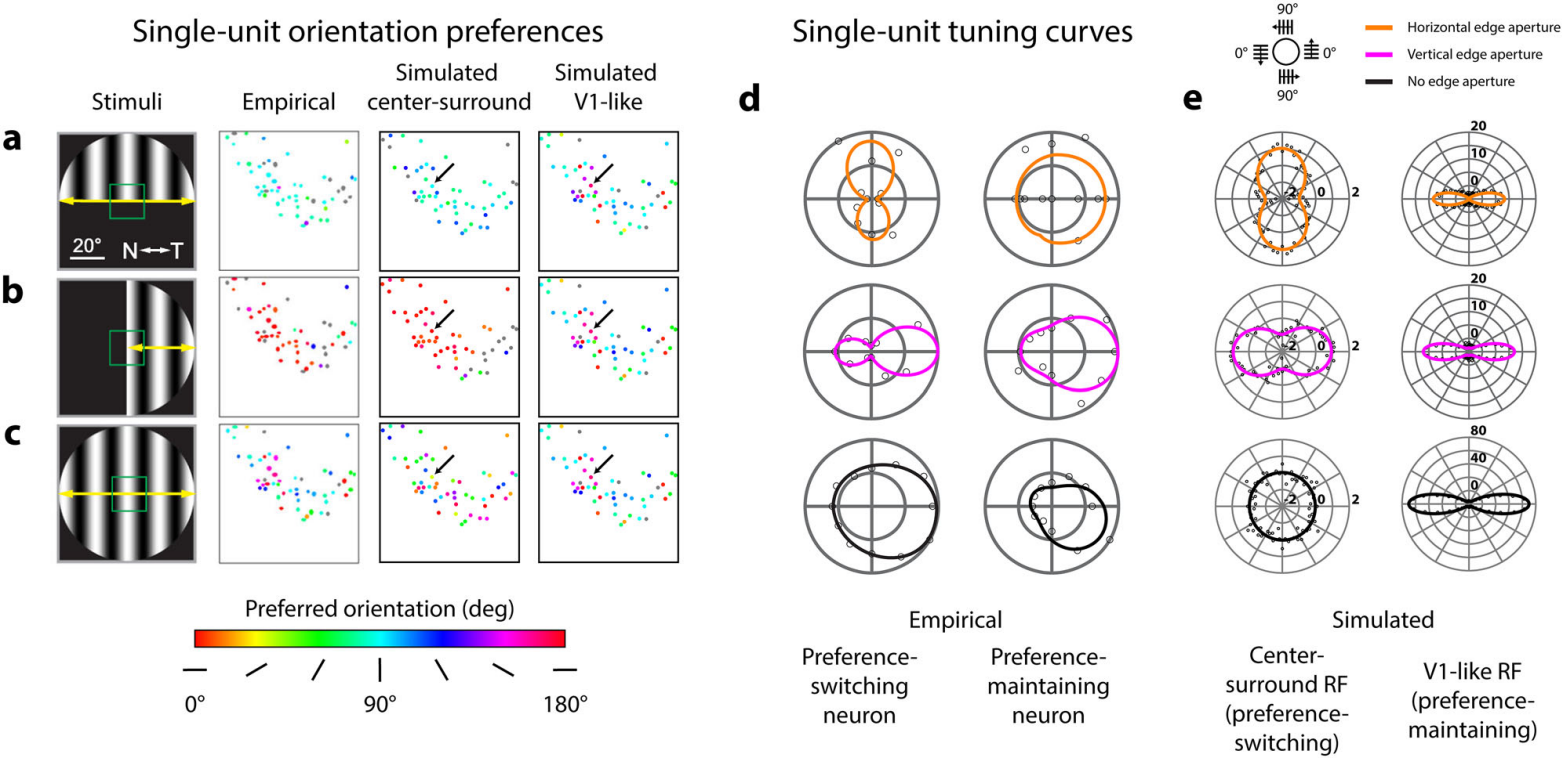
Center-surround and V1-like RF structures produce preference-switching and preference-maintaining tuning patterns. ***a***, Maps of orientation preferences measured empirically from single neurons (middle-left column), simulated using center-surround RF structure (middle-right column), and simulated using V1-like RF structure (rightmost column), in response to grating orientations vignetted by a horizontal edge (leftmost column). Colors correspond to preferred orientation (color scale below panel ***c***). RFs from empirical and simulated neurons fall within the green box shown in visual space (leftmost column); corresponding anatomical locations in either physiological or simulated sSC are depicted in the single-unit maps. Left and middle-left columns adapted from Liang et al. (2023), their Figure 4a–c. ***b***, Orientation preferences as described in ***a***, but with grating stimuli vignetted by a vertical edge. ***c***, Orientation preferences as described in ***a***, but without any edge aperture. ***d***, Empirically measured responses from example preference-switching (left column) and preference-maintaining (right column) neurons to drifting grating orientations (polar plot legend above panel ***e***) vignetted by horizontal, vertical, or no edge apertures (color legend above panel ***e***). Data and visualization adapted from Liang et al. (2023), their Figure 4e,f. ***e***, Simulated responses from example center-surround (left column) and V1-like (right column) RFs to static grating orientations, vignetted by horizontal, vertical, or no edge apertures. Simulated center-surround responses (left column) were generated by the highlighted center-surround unit (***a–c***, middle-right column, black arrows); simulated V1-like responses (right column) were generated by the highlighted V1-like unit (***a–c***, rightmost column, black arrows). Simulated responses from 0–180° grating orientations were duplicated for 180–360° to match ***d***. Units on polar plots correspond to percent change from the mean response across all orientations from the indicated neuron (black arrows, ***a–c***).

Imaged mouse sSC neurons showed a mixture of orientation preference-switching and preference-maintaining properties, which could be modeled by a proportion of V1-like RFs intermingled with center-surround RFs. In the Ca^2+^ imaging data, responses were obtained from a set of 53 neurons at single-cell resolution to oriented gratings vignetted by a horizontal (Fig. 8*a*, far left panel) and vertical aperture (Fig. 8*b*, far left panel), as well as with no aperture (Fig. 8*c*, far left panel). These neurons had RFs centers located within the central 20 degrees of the visual field (Fig. 8*a–c*, far left column, green boxes). The empirical results showed that the set of neurons largely preferred vertical orientations when presented with a horizontal aperture (Fig. 8*a*, middle-left panel), horizontal orientations when presented with a vertical aperture (Fig. 8*b*, middle-left panel), and random orientations when no aperture was present (Fig. 8*c*, middle-left panel). We presented the same stimuli to simulated RFs with RF centers within the same region of visual space and in the same relative spatial configuration as in the imaging data (Fig. 8*a–c*, middle-right and far right columns). Simulated center-surround RFs reproduced the preference-switching effect shown in the imaging data (Fig. 8*a*,*b*, middle-right column) and produced random idiosyncratic orientation preferences due to simulated noise in the absence of stimulus apertures (Fig. 8*c*, middle-right panel). In contrast, simulated V1-like RFs maintained their orientation preferences across aperture conditions (Fig. 8*a,b*, far right column) and did not have their orientation preferences changed by the same amount of simulated noise (Fig. 8*c*, far right panel).

We note that both the empirical and simulated orientation maps do not indicate the degree of orientation selectivity across neurons. In the model, the relative orientation selectivity of the non-aperture condition (Fig. 8*c*) is actually much lower when compared with aperture conditions that produce a reliable, systematic bias for certain grating orientations (Fig. 8*a*,*b*), though this is not explicitly shown as to make a direct comparison to the empirical maps. In the absence of an aperture, the model predicts the exact same response for each stimulus orientation, so the small amount of simulated response noise is the only source of variability in orientation preferences across neurons. To determine whether physiological measurements of population orientation selectivity arise simply due to noise, as predicted by the model, empirical maps should represent not only the preferred orientation, but also the degree of selectivity, e.g., through hue intensity (alpha).

Additionally, we matched the orientation tuning shapes of two types of neurons described in single-unit Ca^2+^ imaging using simulated center-surround and V1-like RFs. The data from Liang et al. (2023) demonstrated examples of neurons that either switched their orientation preference (Fig. 8*d*, left column) or largely maintained their orientation preference (Fig. 8*d*, right column) in response to stimuli of different aperture types. We found that simulated center-surround single-unit RFs produced similar shifts in tuning as the empirically measured preference-switching neurons, in response to apertures of different orientations (compare Fig. 8*d*,*e*, left columns). For simulated V1-like RFs, we found that orientation preferences stayed consistent for the same orientation across aperture types, with their orientation tuning being dictated mainly by the intrinsically oriented structure of the RF (Fig. 8*e*, right column). These V1-like RFs matched the behavior of the empirically measured preference-maintaining neurons (compare Fig. 8*d*,*e*, right columns).

There were also several discrepancies between the tuning shapes from the imaging and simulated data. We note that the empirically derived tuning curves showed a substantial degree of direction selectivity (Fig. 8*d*, note tuning asymmetry). Our simulations aimed to explain how purely spatial changes in RF properties can result in changing orientation preferences. Thus, we explicitly modeled spatial RFs without a temporal component, which cannot capture response differences from motion direction (see Discussion). Another discrepancy was the width of the tuning, or degree of orientation selectivity, between the empirical and the simulated tuning curves, particularly for V1-like RFs (Fig. 8*d*,*e*, right columns). The degree of orientation selectivity was affected by the aspect ratio of the simulated V1-like subregions, and the simulated subregion aspect ratios were based on measurements previously reported in cat striate cortex (Gardner et al., 1999). Further empirical evidence may provide better estimates for what values should be expected for mouse SC.

In summary, these results suggest that center-surround RFs are likely prevalent in mouse sSC as demonstrated by the substantial proportion of neurons shifting their orientation preference in the imaging data. However, the presence of single units that maintained their orientation preference regardless of the aperture suggests a proportion of neurons with V1-like RF structure are intermingled among a larger population with center-surround RF structure.

## Discussion

We show that population models consisting of classic RFs recapitulate stimulus-dependent changes in mouse sSC orientation selectivity (Liang et al., 2023). Simulated orientation preference maps changed with stimulus shape and size, reproducing a wide range of imaging results. The model predicted changes from radial to anti-radial population orientation selectivity with increased spatial frequency, consistent with imaging data. Narrow-field Ca^2+^ imaging at single-cell resolution identified many neurons switching orientation preferences, while some neurons maintained their orientation preferences with changes in stimulus aperture. The model showed the preference-switching subpopulation was better captured with center-surround RF structure and the preference-maintaining subpopulation with V1-like structure. The small preference-maintaining subpopulation may therefore represent a fundamental difference between rodent and primate SC, as V1-like RF structure is generally not reported in primate (Humphrey, 1968; Cynader and Berman, 1972; Goldberg and Wurtz, 1972; but see Chen and Hafed, 2018; Yu et al., 2023). Nonetheless, the modeling demonstrated that all neurons conform to canonical computations well established in early parts of the visual pathway (Enroth-Cugell and Robson, 1966; Movshon et al., 1978; Adelson and Bergen, 1985; Carandini et al., 2005).

Our results demonstrate rewiring of RFs is not required for stimulus-dependent changes in orientation selectivity. Orientation selectivity in V1 arises from precise alignment of feedforward projections of center-surround RFs from LGN to V1 (Hubel and Wiesel, 1962; Reid and Alonso, 1995; Ferster et al., 1996). While recurrent feedback within a circuit (Douglas et al., 1995) may change properties like response gain (Heeger, 1992) or sharpen tuning (Ringach et al., 1997), feedback is not thought to change the intrinsic stimulus preference of neurons. Stimulus-dependent changes in mouse sSC selectivity might appear to be at odds with the notion of fixed connectivity. However, our results demonstrate that stimulus-dependent changes are expected with fixed connectivity, thus reconciling mouse sSC findings with classic neurophysiological results. Changes in selectivity despite fixed connectivity also aligns with theoretical studies (Klein et al., 2014; Fox et al., 2023) explaining apparent shifts in spatial selectivity with attention (Womelsdorf et al., 2006; Anton-Erxleben et al., 2009) and eye movements (Duhamel et al., 1992; Walker et al., 1995; Zirnsak et al., 2014), which also do not depend on dynamic changes in connectivity.

The model based on non-oriented, center-surround RFs can account for numerous reports of mouse sSC orientation selectivity. Common stimuli used to assess orientation selectivity consist of gratings (Wang et al., 2010; Gale and Murphy, 2014; Ahmadlou and Heimel, 2015; Inayat et al., 2015; De Franceschi and Solomon, 2018; Liang et al., 2023) and bars (Feinberg and Meister, 2015; Inayat et al., 2015; de Malmazet et al., 2018; Kasai and Isa, 2022; Li and Meister, 2023). Shown here, both stimuli can produce stimulus-dependent population orientation maps from neurons without intrinsic orientation selectivity. Even full field stimuli without explicit apertures (Wang et al., 2010; Gale and Murphy, 2014; Ahmadlou and Heimel, 2015; Feinberg and Meister, 2015; Inayat et al., 2015; de Malmazet et al., 2018; Kasai and Isa, 2022; Li and Meister, 2023) are susceptible to edge-dependent effects at the edges of the display (compare Fig. 3*a*, square aperture, and Fig. 6). Both radial (Feinberg and Meister, 2015; Liang et al., 2023) and anti-radial (Ahmadlou and Heimel, 2015) orientation preference maps are consistent with the circular-symmetric, center-surround model, depending solely on the combination of the stimulus spatial frequency and the spatial frequency selectivity of the neural population.

While we demonstrate that non-oriented, center-surround RFs are sufficient to produce population orientation selectivity, some evidence suggests there is a small intrinsically orientation selective subpopulation in mouse sSC. Studies have reported orientation selectivity in both amacrine cells (Park et al., 2024; Lei et al., 2024) and retinal ganglion cells (RGCs; Zhao et al., 2013; Baden et al., 2016; Nath and Schwartz, 2017) in mouse retina. Retinal orientation selectivity may arise from dendritic morphology (Nath and Schwartz, 2017) of genetically identified amacrine and RGC subtypes (e.g., B/K wide-field amacrine cells; Park et al., 2024, JAM-B RGCs; Kim et al., 2008). The proportion of RGCs that project to sSC is far higher in mice (85–90%; Ellis et al., 2016) than cats (∼50%; Wässle and Illing, 1980) or primates (∼10%; Perry and Cowey, 1984), so inputs to mouse sSC may include orientation selective RGCs. V1 ablation does not generally affect mouse sSC orientation selectivity (Wang et al., 2010; Ahmadlou and Heimel, 2015; Feinberg and Meister, 2015; Liang et al., 2023), suggesting V1 is likely not a source of selectivity. White noise stimuli and reverse correlation analyses, methods unbiased by stimulus edges (Chichilnisky, 2001), have revealed oriented RF structure in mouse retina (ON and OFF OS RGCs; Zhao et al., 2013; Nath and Schwartz, 2016), LGN (relay cells; Scholl et al., 2013; Zhao et al., 2013), and mouse sSC (Ntsr1+ cells; Li and Meister, 2023). Nevertheless, we have shown that orientation selective population responses are consistent with circular-symmetric RFs and do not require intrinsically selective V1-like neurons.

Extending the model to the temporal domain might allow for an analogous account of direction selectivity. The model used purely spatial RFs designed to explain spatial response properties and therefore cannot explain direction selectivity in mouse sSC (Wang et al., 2010; Gale and Murphy, 2014; Ahmadlou and Heimel, 2015; Inayat et al., 2015; Ito et al., 2017; de Malmazet et al., 2018; Li et al., 2020; Li and Meister, 2023; Liang et al., 2023). Adding temporal frequency selectivity can also account for apparent orientation tuning shifts in population responses (Basole et al., 2003) depending on stimulus size, speed, and direction (Mante and Carandini, 2005). Thus, modeling temporal tuning properties in mouse sSC (Wang et al., 2010; Gale and Murphy, 2014; Ahmadlou and Heimel, 2015; Inayat et al., 2015; Ito et al., 2017; de Malmazet et al., 2018; Li et al., 2020; Li and Meister, 2023; Liang et al., 2023) may provide a more complete explanation of stimulus-dependent selectivity.

Changes in population response with stimulus orientation should not be conflated with the classical definition of orientation selectivity. Hubel and Wiesel described RFs with elongated subfields in V1, responding most strongly to bars of particular orientations. This intrinsic orientation selectivity is a fundamental property of the visual system because it creates an efficient representation of visual inputs (Olshausen and Field, 1996) and arises early in the cascade of visual computations that model the visual system (Yamins et al., 2014). Intrinsic selectivity cannot be inferred simply from observations of population responses varying as a function of stimulus orientation, as neural populations of non-oriented, center-surround RFs can also show stimulus-dependent orientation preferences. Though producing different responses across orientations could loosely be considered a form of orientation selectivity, such a computation is categorically different from the classic observations of orientation selective neurons in V1.

When characterizing orientation selectivity, the choice of stimulus is not the issue, but rather highlights the need for models to interpret results. For example, numerous human fMRI studies have debated the origin and source of orientation selective population responses (Haynes and Rees, 2005; Kamitani and Tong, 2005; Sasaki et al., 2006; Beeck, 2010; Kamitani and Sawahata, 2010; Mannion et al., 2010; Swisher et al., 2010; Freeman et al., 2011, 2013; Alink et al., 2013, 2017; Carlson and Wardle, 2015; Pratte et al., 2016; Wardle et al., 2017; Roth et al., 2018, 2022; Vizioli et al., 2020). Many of these studies have relied on stimulus manipulations, such as blurring edges (Mannion et al., 2010; Freeman et al., 2011; Pratte et al., 2016), using radial spiral stimuli (Alink et al., 2013, 2017; Freeman et al., 2013), and adding grout lines (Alink et al., 2013, 2017), attempting to fix purported stimulus confounds. These manipulations all rely on the assumption that intrinsic orientation selectivity can be revealed from population measurements through the perfect stimulus. However, as the modeling here shows, there is no wrong stimulus with which to study orientation selectivity (bars, apertured gratings, screen edges, or otherwise) because image-computable models operate on any arbitrary stimulus. A model's predicted responses are entirely dependent on the hypothesized neural mechanisms and therefore provide insight into the underlying computations, whether applied to mouse SC, human fMRI, or any other system. Comparison of each model's forward predictions to empirical measurements unambiguously arbitrates between different hypotheses of RF structure (Gardner and Merriam, 2021).

Parsimonious models grounded in interpretable, mechanistic explanations are indispensable for understanding computational principles across model species, though such computations can be implemented in different ways. While increasingly complex computational models (Huth et al., 2012; Yamins et al., 2014) may capture more variance in the data, this increase in variance explained often comes at the cost of interpretability, i.e., clarity in how such models fit the data. The framework we provide demonstrates the utility of classic models built with simple, interpretable components from fundamental principles of neural computation, as these models provide us with strong, general intuitions for how neural systems operate.
